# Bacteria with potential: Improving outcomes through probiotic use following Roux‐en‐Y gastric bypass

**DOI:** 10.1111/cob.12552

**Published:** 2022-09-20

**Authors:** Kylie N. Nowicki, Walter J. Pories

**Affiliations:** ^1^ Brody School of Medicine Greenville North Carolina USA

**Keywords:** bariatric surgery, gastrointestinal microbiome, probiotics, Roux‐en‐Y gastric bypass

## Abstract

Obesity impairs the gastrointestinal microbiome (GM) and may promote micronutrient deficiencies. Bariatric surgery (BS), the most efficacious treatment for severe obesity, produces sustained weight loss and improvements in obesity‐related comorbidities, but might not fully restore microbial balance. Moreover, BS may result in deleterious consequences that affect weight loss and further intensify post‐operative micronutrient deficiencies. To date, the use of probiotics appears to be associated with greater weight loss in bariatric patients, improved vitamin synthesis and availability, and decreased instances of small intestinal bacterial overgrowth. Thus, manipulation of the GM through probiotics represents a promising therapeutic approach in bariatric patients. This review aims to highlight the benefits of using probiotics in bariatric surgical patients by addressing the impact of probiotics on the GM, how BS impacts the microbial environment, associations between gastrointestinal dysbiosis and negative health outcomes, how BS contributes to dysbiosis, and how probiotics may prove efficacious in treating patients who undergo Roux‐en‐Y gastric bypass (RYGB). Based on currently available data, the role of microbial manipulation post‐RYGB through probiotics has shown great potential, but a further clinical investigation is warranted to better understand their efficacy.

**Highlights**

- Alterations in gastrointestinal microbiome (GM) composition are related to many disease states, including obesity.
- Dysbiotic changes in GM have been linked to various complications following bariatric surgery (BS).
- Probiotic use post‐operatively may help mitigate maladaptive complications of Roux‐en‐Y gastric bypass.
- More research is needed on restoring the GM to improve clinical outcomes post‐BS.

## 1 INTRODUCTION

The gastrointestinal microbiome (GM) plays a vital role in modulating host health. The metabolic activity of the microbiome equates to that of a distinct organ through its establishment of a symbiotic relationship with the host and its regulation of several physiological pathways.^1^

Intestinal microbes have been implicated in regulating gastrointestinal motility, intestinal barrier homeostasis, vitamin synthesis, nutrient absorption and fat distribution. They also significantly influence the metabolism, physiology and immune development and function of the host.^2^

Of interest are the effects of gut microbes on energy metabolism and their influence on the pathogenesis of metabolic diseases. Normally, microbes in the host protect from pathogenic colonization; however, certain disease states show marked phenotypic differences in microbial composition, reflecting a dysbiosis between the microbes and host.^3^

Such microbial alterations have been observed in many disease states, including cardiovascular disease (CVD), cancer, malignancy, type 2 diabetes mellitus (T2DM), nonalcoholic fatty liver disease (NAFLD), obesity, psychiatric and inflammatory disorders, disorders of the gut–brain axis and numerous immune disorders.^4^

This review highlights the characteristic microbial alterations of individuals with obesity, how gastric bypass modifies the microbiome, the efficacy of using probiotics to mitigate microbial dysbiosis, and proposes further areas of research for treating patients post‐bariatric surgery (post‐BS).

While there are various bariatric surgical techniques, this review focuses on the Roux‐en‐Y gastric bypass (RYGB), due to the substantial impact it has on the microbiome. In comparison to more conservative techniques such as the gastric sleeve, the RYGB entails dramatic anatomic rearrangement of the digestive tract. Such changes have been shown to not only affect nutrient absorption and microbial distribution throughout the intestine but also alter the metabolic function and hormonal regulation. These aspects of the RYGB make it an intriguing model for studying the effects of microbial alterations on the host and the opportunities for implementing probiotics as an adjunctive treatment.

### 1.1 Search criteria

A literature search was conducted in June 2020 using PubMed (MED‐LINE), EMBASE, and Google Scholar databases. Keywords included the MeSH terms: ‘probiotics’, ‘gastric bypass’ and ‘bariatric surgery’ along with conceivable synonyms. Given the dearth of articles generated that applied to our research, additional keywords and their synonyms relating to the ‘gastrointestinal microbiome’ were used to increase the sensitivity of the search. Only articles written in English and for which the full text was available were selected. Original research and review articles on both animals and humans were included, and there were no limitations regarding the period of publication to maximize understanding of the knowledge‐to‐date. Twenty‐seven articles were initially selected, and additional publications of relevance were identified by hand‐searching the reference lists of eligible articles. The literature search was repeated in January 2022 to identify any new articles pertinent to our research, of which none were identified.

## 2 BACKGROUND INFORMATION

### 2.1 The microbiome and obesity

The gastrointestinal tract contains over a thousand distinct microbial species and more than three million genes, compared to approximately 30 000 in the human genome.^5^ This microbiota has a symbiotic relationship with the host, modulating inflammation and the immune system; acting in the biotransformation of xenobiotics and the absorption of micronutrients; synthesizing vitamins, enzymes and proteins used by the host; fermenting energetic substrates; providing resistance to pathogens; and changing energy availability in the diet.^5^, ^6^

A healthy GM can adapt to various internal and external factors by modifying to meet human needs and maintain a balanced mutualistic relationship with the host.^1^ For example, diet plays a fundamental role in shaping the composition of the GM by modulating the amount and the diversity of substrates that facilitate bacterial development.^1^ In turn, this shapes GM composition and determines whether the most efficient nutrient‐competing bacteria will predominate.^1^

Interruption of the symbiotic relationship between microbes and host results in gastrointestinal dysbiosis and may contribute to obesity and its consequences: T2DM, NAFLD, CVD and cancer.^1^ However, dietary interventions aimed at combatting obesity often have harmful repercussions on GM. For example, restrictive diets promote a reduction in GM diversity and correlate with macronutrient deficiency rather than weight loss.^1^

Patterns of phenotypic variance have been identified in overweight populations, which may exacerbate the severity of the disease state. For example, low microbial gene richness (MGR) is more prevalent in severe obesity, as compared to lean or overweight/moderate obesity.^2^, ^3^ This loss of MGR is associated with several pathogenicities, such as insulin resistance, chronic inflammation and metabolic disturbances.^1^ Moreover, obesity is accompanied by vitamin and mineral deficiencies that further alter and impair the GM.^7^

Several mechanisms to explain microbial influence on obesity have been proposed, including (1) short‐chain fatty acid production, (2) regulation of food intake, (3) nutrient absorption, (4) circulation of microbe‐derived enterotoxins, (5) reduced angiopoietin production and (6) peripheral control of circadian rhythm.^8^

T2DM development in individuals with obesity is particularly concerning. These patients possess a marked gastrointestinal dysbiosis, characterized by a decrease in the Bacteroidetes/Firmicutes ratio and functional bacteria, with an increase in various opportunistic pathogens and some endotoxin‐producing Gram‐negative bacteria that alter host energy metabolism.^9^ Moreover, the accumulation of gut‐derived bacterial inflammatory molecules might hasten inflammation in T2DM.^9^

### 2.2 Bariatric surgical intervention

BS is arguably the most efficacious treatment for severe obesity. Candidates often have markedly impaired nutritional status, likely related to poor‐quality food choices that provide inadequate amounts of vitamins and minerals despite higher total caloric intake. These poor eating habits, along with other factors such as chronic diseases, medication, and so forth, contribute to intestinal dysbiosis.

The RYGB is a surgical procedure proven to treat both severe obesity and T2DM. Although the mechanism by which RYGB improves various disease states is still under investigation, rearrangement of the intestinal microbiota may contribute to the improvement of obesity‐related chronic inflammation.^1^ However, complications may occur due to the anatomic and physiological changes, which themselves are risk factors for altering the intestinal microbiota. Such risk factors include reflux, vomiting, electrolyte and nutritional abnormalities, intestinal dysmotility, lower secretion of gastric acid and displacement of typical bacteria from the small intestine.^10^ Inadequate digestive enzyme secretion following gastric bypass might also cause inadequate digestion of fats and proteins.^1^ Furthermore, bacterial overgrowth (BO) and increased intestinal permeability may contribute to ongoing inflammation.^11^ Finally, maladaptive changes in the intestinal flora may lead to vitamin deficiencies, alterations in fat absorption, and malnutrition post‐surgery.^1^

Since many of these negative effects are linked to dysbiotic modifications in the GM, researchers have been investigating probiotics as a method of mitigating these changes and promoting a better quality of life (QoL) in patients post‐BS.^12^, ^13^

Studies indicate that probiotic use prevents and treats various health disorders, such as gastrointestinal infections, inflammatory intestinal disease, lactose intolerance and many kinds of cancer; and reduces the collateral effects of antibiotic therapy.^14^ With obesity, patients undergoing RYGB are ideal for studying probiotics' effects on the microbiota because the neutral environment of the gastric pouch, following surgery, will not destroy probiotics as a normally acidic, the undivided stomach might.^13^

This population is uniquely suited for investigating probiotic utilization in combatting intestinal disorders and has the potential to see marked improvements through their use.

### 2.3 Current understanding of the microbiome

The gut microbiota includes bacteria, fungi, archaea, protozoa and viruses that interact with the host and each other to affect the host's physiology and health.^15^ The collective genome of the bacterial component (microbiome) is 150‐fold larger than the human genome.^15^ These bacteria are from ~500 species, with 99% belonging to four main families: Firmicutes (64%), Bacteroidetes (23%), Proteobacteria (8%) and Actinobacteria (3%).^15^

Gastrointestinal bacteria play significant roles in human health, including vitamin synthesis, improvements in digestion and promotion of angiogenesis and nerve function.^6^ The microbiota is also involved in harvesting energy from the diet through the utilization of indigestible compounds, micronutrient absorption, xenobiotic biotransformation, immune system stimulation and pathogen resistance.^6^ However, alteration of GM composition can cause multiple diseases in humans and animals.^6^ Studies have investigated methods of regulating microbial dysbiosis to treat different disease states, with probiotics being an efficacious intervention for promoting beneficial microbial growth and balance.^14^, ^16^, ^17^

### 2.4 Probiotic impact on the microbiome

According to the World Health Organisation, probiotics are ‘live microorganisms that, when administered in adequate amounts, confer a health benefit on the host’.^18^ A good probiotic candidate is a bacterial strain with characteristics that contribute to indigenous colonization, such as tolerance to low pH, resistance to bile salts and adhesion to the host epithelium.^19^

Probiotics, either colonizing or in transit, have various effects on the host's internal environment, such as (1) modulation of endogenous microbacterial functions and competitive exclusion of pathogens, (2) enhancement of gut epithelial barrier function, cytokine production, inflammation and other innate immune responses, (3) influence on the host's metabolic function, (4) integration of peripheral and central food intake signals and (5) regulation of weight gain.^20^

Interaction of probiotic bacteria with host epithelial cells may also promote phenotypic changes in the bacteria that improve their mutualistic relationship with the host.^21^

Clinically, probiotics have proven effective for treating infective gastroenteritis, antibiotic‐associated diarrhoea, irritable bowel syndrome, ulcerative colitis, general inflammation, allergies, pancreatitis, Crohn's disease and many other disease states.^6^, ^13^, ^14^

While some studies have failed to show that probiotics affect the composition and diversity of the main bacterial populations, others have reported wider changes in microbial composition with probiotic administration.^22^ Furthermore, even when a change in the overall microbial composition is not proven, other changes—such as in mucin layer depth, antimicrobial peptide production, increased abundance of beneficial bacterial species, or changes in metabolite synthesis—could influence obesity and metabolic alterations.^14^, ^17^, ^22^

### 2.5 Strain‐specific interactions

While probiotics are clinically beneficial in treating various disease states, the biological effects are largely strain‐dependent: not every probiotic will perform the same role.^17^

For instance, Lactobacilli are more highly concentrated in the small intestine, where they produce acid, reduce pH and prevent the growth of pathogenic bacteria.^16^ As such, they are linked to immunomodulatory effects.^16^ On the other hand, Bifidobacteria are more prevalent in the large intestine, and a reduction results in gastrointestinal disorders, such as constipation.^23^

Understanding GM's composition is necessary to appreciate the clinical efficacy of probiotics. The bacterial species found in the human GM primarily consist of Firmicutes (Ruminococcus, Clostridium and Eubacteria), Bacteroidetes (Porphyromonas, Prevotella), Proteobacteria and Actinobacteria (Bifidobacterium).^6^, ^15^ Lactobacilli, Streptococci and *Escherichia coli* are found in smaller numbers, but nonetheless have vital roles in maintaining host homeostasis.^6^

Studies indicate that individuals with obesity have a reduction in the Bacteroidetes/Firmicutes ratio, possibly due to an increase in Firmicutes.^24^ This phenotypic alteration provides genetic material for an increased capacity to harvest energy from the diet.^25^, ^26^

Knowing that the effects of bacteria in the microbiome are largely strain‐specific enables researchers to use targeted probiotics to treat various diseases.

## 3 GASTRIC BYPASS INDUCED MICROBIAL ALTERATIONS

### 3.1 Changes in GM composition

Through anatomic and physiological alterations in the gastrointestinal tract, BS induces changes in the composition of the microbiome. Early changes occur under the same time frames as improvements in glucose tolerance and reduced insulin resistance.^27^ In contrast, body weight and adiposity changes occur over weeks and months.^27^ These results suggest microbial alterations may be involved in resetting metabolic set points that are distinct from adiposity.^27^, ^28^

Liou et al. found that RYGB increases the Bacteroidetes/Firmicutes ratio, while also increasing *E. coli* and Verrucomicrobia as a relative percentage of the microbial community.^28^

In addition, Furet et al. found that following RYGB: (1) the Bacteroides/Prevotella group increased and was negatively correlated with corpulence, (2) *E. coli* species increased and inversely correlated with fat mass and leptin levels independently of changes in food intake and (3) lactic acid bacteria and Bifidobacterium decreased.^29^

In agreement with Bajzer and Seeley, these changes in gut microbiota post‐RYGB could reflect the maximization of energy harvest as an adaptation to the starvation‐like conditions induced by BS.^29^, ^30^ Interestingly, most bacterial changes stabilized 6 months following surgery, while corpulence and metabolic factors continued to improve, favouring this interpretation.^29^

Other anatomic and physiological changes resulting from RYGB further modify GM composition. For example, RYGB reduces stomach size and rewires the small intestine, allowing food to bypass the distal stomach, duodenum and proximal jejunum. These modifications decrease the time food encounters microbial species in these locations and alters the nutrient availability for this microbiota.^31^

Among these anatomic modifications, the smaller gastric pouch and formation of a gastrojejunostomy favour the presence of oxygen in this part of the intestine and the development of facultative anaerobic species.^29^ Furthermore, pH increases due to the lower secretion of gastric acid, which can hinder the development of Lactobacilli and Bifidobacteria, and modify the oxidoreduction potential in the gut, likely affecting aerobes and facultative aerobic microorganisms.^32^

Changes in intestinal colonization following BS may also result from alterations in eating habits post‐surgery, with the reduction of fat intake, augmentation of polysaccharides and fluctuations in intestinal acidity influencing nutrient bioavailability.^5^ Finally, medication might play a role, as the use of proton‐pump inhibitors after BS increases the proportion of Firmicutes.^33^

### 3.2 Microbial gene richness

Aron‐Wisnewsky et al.^3^ observed that RYGB increases MGR. Most patients before surgical intervention had low MGR, which correlated with increased trunk‐fat mass and comorbidities (T2DM, hypertension and severity). RYGB increased MGR 1‐year post‐surgery and was associated with metabolic improvements.^3^, ^34^ MGR was inversely correlated with fat mass, leptin, fasting insulin, insulin resistance, triglyceride levels and systemic inflammation.^3^, ^34^ MGR was also negatively correlated with glucose intolerance and subcutaneous adipocyte volume, while positively associated with adiponectin.^3^, ^34^ However, MGR was not fully corrected following surgery, indicating that additional interventions aimed at further restoring microbial balance may be warranted.^3^

### 3.3 Metabolic alterations

Studies have shown that RYGB is associated with substantial functional changes in the GM.^35^ Decreases in microbial functions, such as pathways related to carbohydrate fermentation, the citrate cycle, glycosaminoglycan degradation and lipopolysaccharide synthesis, were observed post‐surgery, as a microbial enzymatic activity more closely resembled lean controls.^35^

Despite dramatic weight loss and improvement in metabolic markers, MGR is not fully corrected by RYGB.^3^ In addition, alterations in the GM and the anatomic and physiological changes resulting from BS can cause nutrient deficiencies and other medical complications.^1^

RYGB affects vitamin and mineral absorption by excluding the remnant stomach and upper part of the small intestine from gastrointestinal transit.^36^, ^37^ Impairments in micronutrient absorption occur, since the food bolus is not exposed to biliopancreatic secretions, and bypass of the distal stomach decreases the output of gastric juices, further impeding micronutrient absorption.^36^, ^37^

The main micronutrient deficiencies reported after RYGB include: vitamin B12, folic acid, iron, thiamine, vitamin D and calcium.^1^ Other reported nutritional deficiencies following weight loss surgery, particularly mixed bariatric procedures, include liposoluble vitamins and minerals such as copper, zinc and selenium.^1^

Kyoto Encyclopedia of Genes and Genomes orthologs and pathway analysis has also noted changes in gut microbial metabolism associated with the transport of other essential nutrients, such as manganese, carbohydrate utilization, amino acid uptake, and purine and fatty acid metabolism.^1^

Thus, in addition to the mechanical restriction of caloric intake, RYGB also impairs macro‐ and micronutrient absorption.^31^

Furthermore, these nutrient deficiencies can exacerbate maladaptive GM compositional changes post‐BS. Lack of vitamin A increases the proportions of *Bacteroides vulgatus*, while the shortage of vitamins C and E significantly inhibits Bacteroides species.^38^, ^39^ Proportions of Bacteroidetes and Firmicutes are also severely reduced by a lack of dietary components with antioxidant properties, leaving room for facultative anaerobes to flourish, such as *E. coli*, or pathogens like Shigella and Salmonella.^40^

This increase in facultative anaerobes makes bariatric patients more likely to develop small intestinal bacterial overgrowth (SIBO), and excessive growth of atypical bacteria which compete with the host for nutrients, while resultant metabolites cause mucosal injury.^1^ SIBO further complicates weight loss, increases inflammation and the risk of micronutrient deficiencies, and causes physical side effects, including abdominal pain, bloating and diarrhoea.^41^ Sabate et al.^42^ found that SIBO was present in 15% of patients before RYGB and increased up to 40% post‐surgery, with heightened susceptibility likely due to nutrient deficiencies, mechanical stasis, and the creation of blind loops from surgery.

Other surgical complications resulting in nutrient deficiencies include recurrent nausea and vomiting and food intolerances.^43^

The micronutrient status of bariatric patients may further deteriorate post‐RYGB, affecting GM composition.^1^, ^3^ Furthermore, since low MGR is not completely reversed after BS, specific interventions aimed at fully restoring microbial balance and improving GM–host interactions are needed after surgical intervention.

### 3.4 Probiotics' efficacy post‐BS

Studies reflect GM involvement in inflammation and other physiologic complications following BS.^3^, ^5^, ^29^ Thus, using microbial regulators, such as probiotics, could benefit patients undergoing surgery.^12^, ^13^, ^44^ Research supports the modulating effects of probiotics on endotoxins, inflammatory and oxidative stress status and improvements in micronutrient levels.^44^

Probiotics have been shown to accentuate weight loss, increase food tolerances and improve QoL in patients post‐RYGB.^12^, ^44^ Chen et al.^12^ found that probiotic and digestive enzyme therapy reduced instances of excessive flatulence, belching, heartburn, bloating and pain in BS patients. In addition, Woodard et al.^13^ observed a significant reduction in BO and improved post‐operative vitamin B12 levels with probiotic use. Furthermore, certain species of Lactobacillus and Bifidobacterium can produce vitamins such as folates and vitamin B12 and may influence vitamin D status.^1^, ^44^

While these findings have been confirmed by other studies, Mokhtari et al. found that probiotics may be unable to produce long‐lasting effects, while other studies have shown conflicting evidence for their efficacy in preventing SIBO, improving QoL, and maintaining weight loss.^1^, ^5^, ^44^ Therefore, more research is needed to further identify how probiotic treatment post‐BS may prove efficacious for patients.

## 4 LIMITATIONS

Deciphering the immediate and long‐term consequences of BS is challenging since these procedures cause numerous physiologic and metabolic adaptations. Variations in bariatric surgical techniques further complicate the analysis, as modifications in the location and length of anastomoses during gastric bypass may uniquely alter the microbiome. In addition, studies vary in their designs used to assess probiotic efficacy, interventions and observational periods, and the diversity of probiotic strains utilized.

There is also extensive individual variation in the composition of the intestinal microbiota, and sampling techniques occur mainly through stool samples, making it difficult to discern the location of different microbial species within the gastrointestinal tract. Thus, the microbial composition and response to probiotic supplementation may differ according to the characteristics of each individual.

## 5 CONCLUSION

Obesity dramatically alters GM and augments micronutrient deficiencies. BS has significant impacts on weight loss, improves obesity‐related comorbidities, and substantially changes GM composition, but alone it might not fully restore microbial balance. Moreover, deleterious consequences from BS affect the weight loss and micronutrient status of patients. Pre‐operative micronutrient deficiencies and failure to properly supplement patients post‐surgery may further intensify micronutrient deficiencies. Many of these complications have been linked to dysbiosis in the microbiome following BS and may play a role in poor health outcomes following surgery.

These observations highlight the potential benefit of probiotic administration to improve GM balance and mitigate many of the negative consequences of surgical intervention. In particular, the physiologic and mechanical alterations induced through RYGB may serve as the prime vehicle for implementing and observing the influence of probiotics on patient outcomes post‐surgery. A perspective of this work is to use strategies aimed at restoring the GM ecosystem before, during, or after BS and to examine whether these interventions could further improve MGR and/or clinical outcomes post‐surgery.

### 5.1 Future areas for research

Probiotic use in bariatric surgical patients is promising, but more research is needed to better understand their utility.

With stark distinctions between obese and lean GM profiles, research should focus on defining what constitutes a healthy microbial balance and investigating strain‐specific interactions on differing phenotypes.

Investigation into the microbial alterations resulting from other bariatric surgical techniques, such as the gastric sleeve, and subsequent comparison to the RYGB, may prove beneficial in determining how intestinal manipulation affects microbial balance and the host metabolic state.

Research should also attempt to determine whether unique GM profiles correlate with certain disease states. Such research could then promote the supplementation of strain‐specific probiotics to manage various health conditions.

Supplementing probiotics has the potential to benefit many individuals with metabolic diseases. However, more research is needed on their safety and efficacy for use in certain populations, such as those with compromised immune systems, who may be negatively affected by the consumption of bacterial strains.

As more information is obtained on the role of probiotics in human health, research should further investigate the role of prebiotics in modulating GM; recent studies on prebiotics have shown promise in improving intestinal microbial balance, but more information is necessary.

Antibiotic use in mitigating microbial dysbiosis may also prove efficacious, once maladaptive bacterial profiles are identified.

Finally, with the increased use of probiotics in clinical practice, heightened FDA regulation is warranted to avoid challenges with quality control.

## CONFLICT OF INTEREST

The authors declare no conflict of interest.
